# Red Bumps, Red Flags: A Case Series of Four Neoplasms Masquerading as Inflamed Cutaneous Cysts

**DOI:** 10.7759/cureus.102703

**Published:** 2026-01-31

**Authors:** Jared Hensley, Smaran Marupudi, Kelly Maedo, Saba Suleman, Eric Sandrock

**Affiliations:** 1 Dermatology, University of Texas Rio Grande Valley School of Medicine, Edinburg, USA; 2 Dermatology, HCA Healthcare Corpus Christi Medical Center-Bay Area Program, McAllen, USA

**Keywords:** amelanotic melanoma, dermatofibroma, dermatofibrosarcoma protuberans, epidermal inclusion cyst, undifferentiated pleomorphic sarcoma

## Abstract

Red, inflamed cutaneous nodules are frequently diagnosed as epidermal inclusion cysts in both primary care and dermatology settings. However, several benign and malignant entities can closely mimic an inflamed cyst, leading to delayed diagnosis and suboptimal management. We present four patients who initially presented with erythematous nodules clinically suspected to be cysts; final histopathologic diagnoses included dermatofibroma, amelanotic melanoma, dermatofibrosarcoma protuberans, and undifferentiated pleomorphic sarcoma. For each case, we describe the clinical presentation, histopathology, and management. The presence of a central punctum and superficial mobility strongly favors an epidermal inclusion cyst. In contrast, the absence of a punctum, rapid growth, deep fixation, a positive dimple sign, or atypical dermoscopic vascular patterns should raise suspicion for a neoplasm. Final diagnosis is based on histopathologic examination, which guides subsequent treatment in accordance with current guidelines. This series underscores the importance of maintaining a broad differential diagnosis for erythematous nodules and obtaining histopathologic confirmation when clinical features are atypical. Early recognition and appropriate biopsy technique may improve patient outcomes.

## Introduction

Patients commonly present to the clinic with an "abscess" or a bump under the skin. They frequently report that the area is painful and growing, and that they tried to "pop" the lesion with variable success in expressing material. It is easy to assume these bumps are epidermal inclusion cysts (EICs), sometimes called sebaceous cysts, particularly in a primary care setting. However, several benign and malignant entities can closely mimic an inflamed cyst, leading to delayed diagnosis and suboptimal management [1-3].

EICs rank among the most common benign cutaneous lesions encountered in clinical practice. They typically present as subcutaneous nodules with a characteristic central punctum and free mobility [1]. When inflamed or ruptured, EICs become erythematous and tender, closely resembling neoplastic processes. Accurate diagnosis depends on careful physical examination, including assessment for a central punctum or dimple sign, supplemented by dermoscopic evaluation and appropriate biopsy technique [2,4]. Misdiagnosis can result in delayed treatment, inadequate surgical margins, and worsened prognosis, particularly for aggressive malignancies such as amelanotic melanoma or high-grade sarcomas [5,6].

This case series presents four patients whose lesions were initially thought to be cysts but were ultimately diagnosed with distinct entities requiring different therapeutic approaches: dermatofibroma, amelanotic melanoma, dermatofibrosarcoma protuberans (DFSP), and undifferentiated pleomorphic sarcoma (UPS). We emphasize clinical and dermoscopic clues that help differentiate these neoplasms from EICs and outline current guideline-based management strategies.

## Case presentation

Case 1: Dermatofibroma

**Figure 1:**
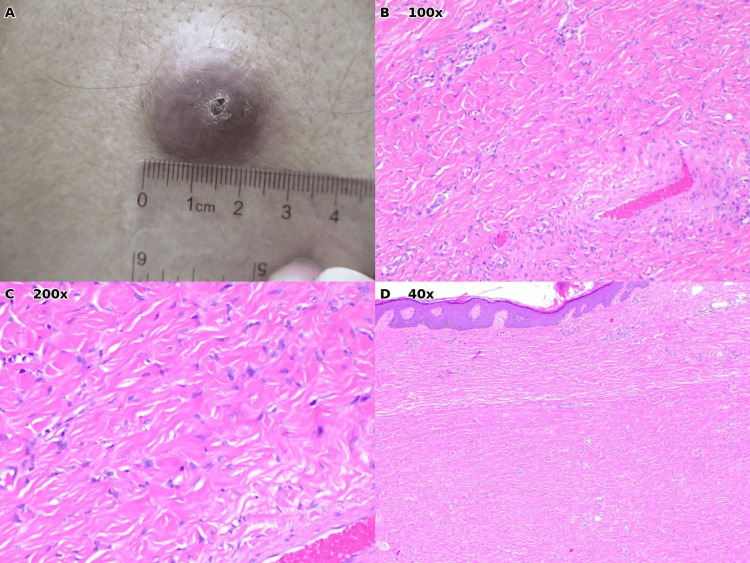
Case 1: Dermatofibroma of the left lower extremity. (A) Clinical photograph demonstrating a firm, well-circumscribed, brown-red nodule measuring approximately 2 cm with ruler for scale. (B) Histopathology (H&E, ×100) showing dermal spindle cell proliferation arranged in a storiform pattern. (C) Higher magnification (H&E, ×200) demonstrates the characteristic pinwheel arrangement of uniform spindle cells with elongated nuclei. (D) Low-power view (H&E, ×40) illustrating overlying epidermal hyperplasia (acanthosis) and peripheral collagen trapping, classic features of dermatofibroma.

A 71-year-old male presented with a firm, well-circumscribed, brown-red nodule on the left lower extremity measuring approximately 2 cm in diameter (Figure 1A). The lesion was clinically diagnosed as an epidermal inclusion cyst, and excision was performed.

Histopathologic examination revealed findings diagnostic of dermatofibroma: a dermal proliferation of bland spindle cells arranged in a storiform (pinwheel or cartwheel) pattern (Figures 1B, 1C). Additional characteristic features included overlying epidermal hyperplasia (acanthosis) and peripheral collagen trapping, where the tumor appears to engulf pre-existing dermal collagen bundles at its edges (Figure 1D). These histologic findings, particularly the storiform architecture and collagen trapping, are hallmarks that distinguish dermatofibroma from other spindle cell neoplasms [2]. Dermatofibromas classically occur on the extremities and may be mistaken for cysts when they present as firm, slightly raised nodules; however, unlike EICs, they typically lack a central punctum and demonstrate a characteristic "dimple sign" when lateral pressure is applied [2,7]. Given the complete excision and benign diagnosis, no further treatment was required.

Case 2: Amelanotic melanoma

**Figure 2:**
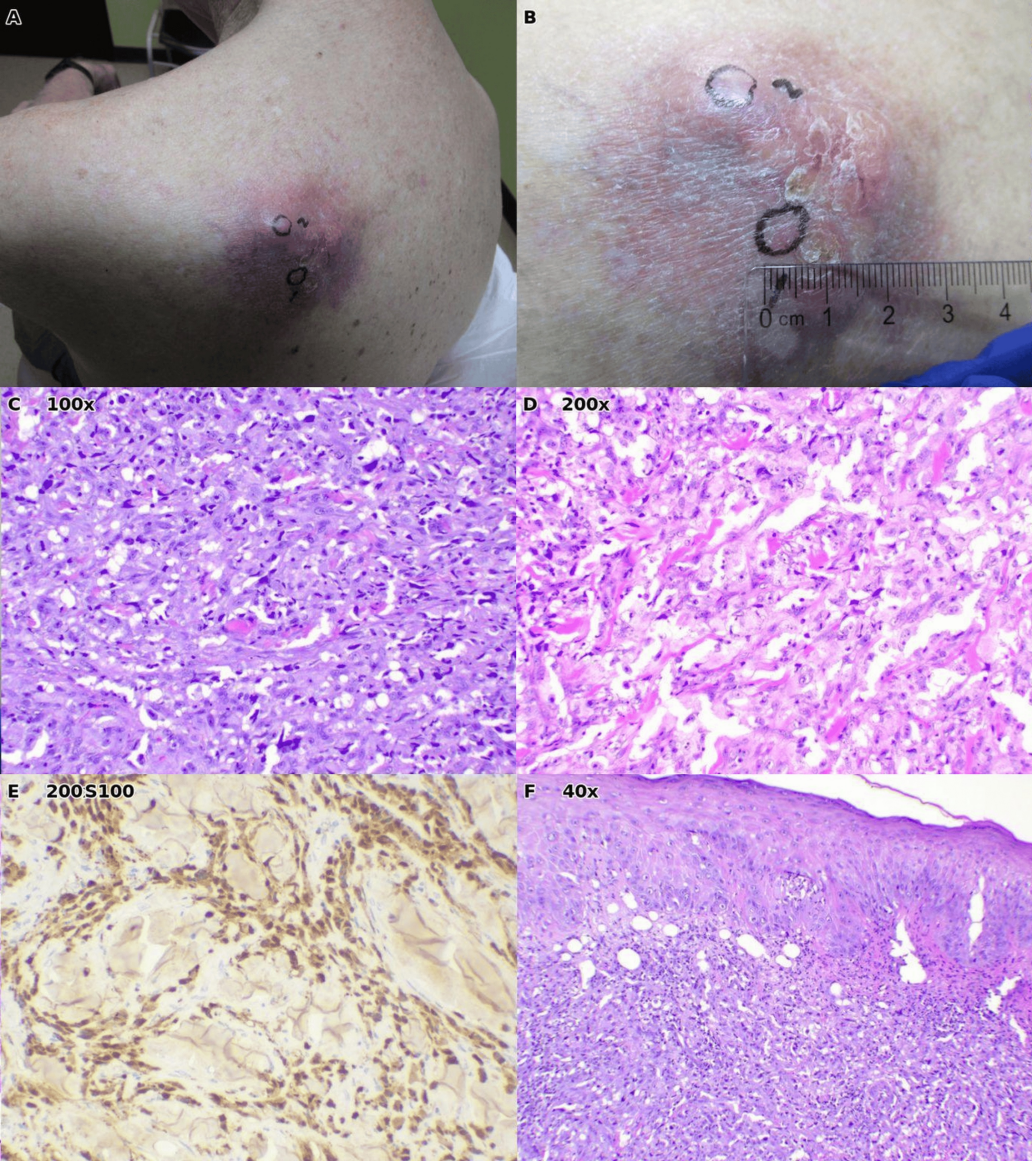
Case 2: Amelanotic melanoma of the left upper back. (A) Clinical photograph showing an irregular, erythematous mass with surrounding inflammation. (B) Additional clinical view with ruler demonstrating size and irregular surface. (C) Histopathology (H&E, ×100) reveals a densely cellular atypical neoplasm infiltrating the dermis. (D) Higher magnification (H&E, ×200) showing pleomorphic tumor cells with prominent nuclei and absent melanin pigmentation. (E) Immunohistochemical stain (×200). (F) Low-power view (H&E, ×40) illustrating depth of invasion into subcutaneous adipose tissue.

An 84-year-old male with a history of melanoma in situ, treated in February 2024, was seen for follow-up in March 2024 and was scheduled to return in November 2024. The patient, a winter Texan, planned to see oncology in the northern United States over the summer. In January 2025, the patient presented with a large mass on the left upper back that had developed over approximately two months (Figures 2A, 2B). The rapid growth and clinical appearance raised immediate concern for malignancy rather than a benign cyst.

Two punch biopsies were performed due to the lesion's size and heterogeneity. Histopathologic examination demonstrated a malignant epithelioid neoplasm characterized by sheets of atypical cells with marked nuclear pleomorphism, prominent nucleoli, and frequent mitotic figures (Figures 2C, 2D). Notably, the tumor lacked melanin pigment, a feature that makes amelanotic melanoma particularly challenging to diagnose clinically as it lacks the brown-black coloration typically associated with melanoma [4]. The neoplasm showed extensive invasion into the subcutaneous adipose tissue (Figure 2F). The final pathologic interpretation favored dedifferentiated melanoma, consistent with the patient's history of prior melanoma in situ. The patient was referred to a tertiary care center for definitive management. Treatment records were not available for review, and the patient did not return to our clinic for follow-up.

Case 3: Dermatofibrosarcoma protuberans

**Figure 3:**
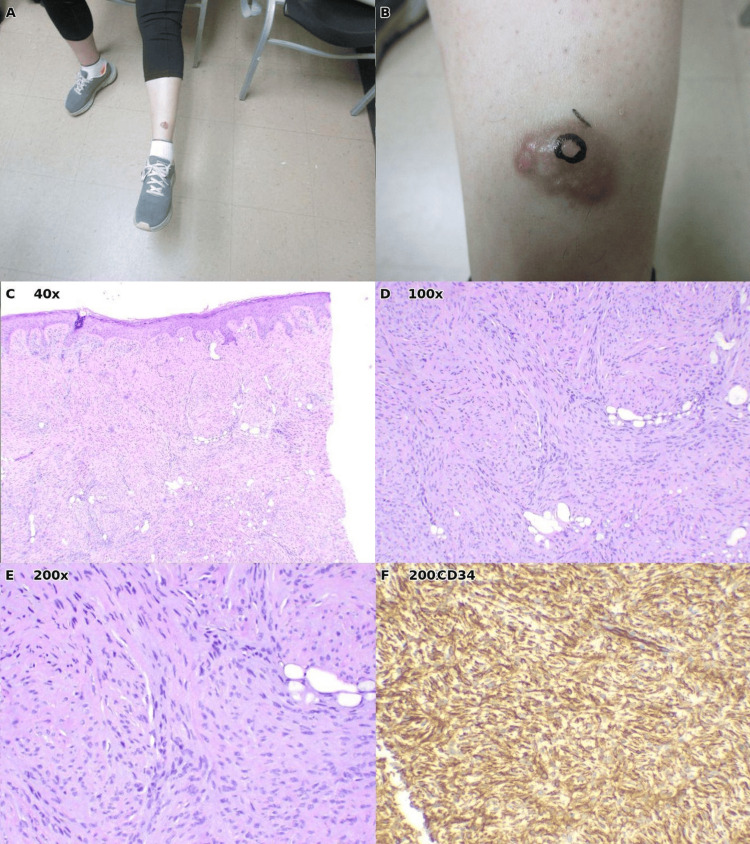
Case 3: Dermatofibrosarcoma protuberans of the left shin. (A) Clinical photograph of the patient's lower extremities, showing a lesion on the left anterior leg. (B) Close-up view demonstrating a firm, flesh-colored to erythematous protuberant nodule. (C) Low-power histopathology (H&E, ×40) showing storiform spindle cell proliferation with a characteristic honeycomb infiltration pattern, where tumor cells surround individual adipocytes in the subcutis. (D) Intermediate magnification (H&E, ×100) demonstrating uniform spindle cells infiltrating around fat lobules. (E) High-power view (H&E, ×200) showing monotonous spindle cells with elongated nuclei arranged in fascicles. (F) Immunohistochemical stain (×200).

A 28-year-old female presented with a firm plaque on the left shin that had evolved into a protuberant nodule over time (Figures 3A, 3B). The lesion displayed reddish discoloration and induration.

A punch biopsy, including the subcutaneous layer, was performed. Histopathologic examination revealed a storiform spindle cell proliferation composed of monotonous, slender spindle cells with minimal cytologic atypia arranged in intersecting fascicles (Figures 3D, 3E). The diagnostic hallmark was the infiltration pattern into the subcutis: tumor cells surrounded individual adipocytes in a characteristic "honeycomb" or "lace-like" pattern (Figure 3C). The patient underwent Mohs micrographic surgery with complete margin clearance. At the last follow-up in October 2025, there was no evidence of recurrence.

Case 4: Undifferentiated pleomorphic sarcoma

**Figure 4:**
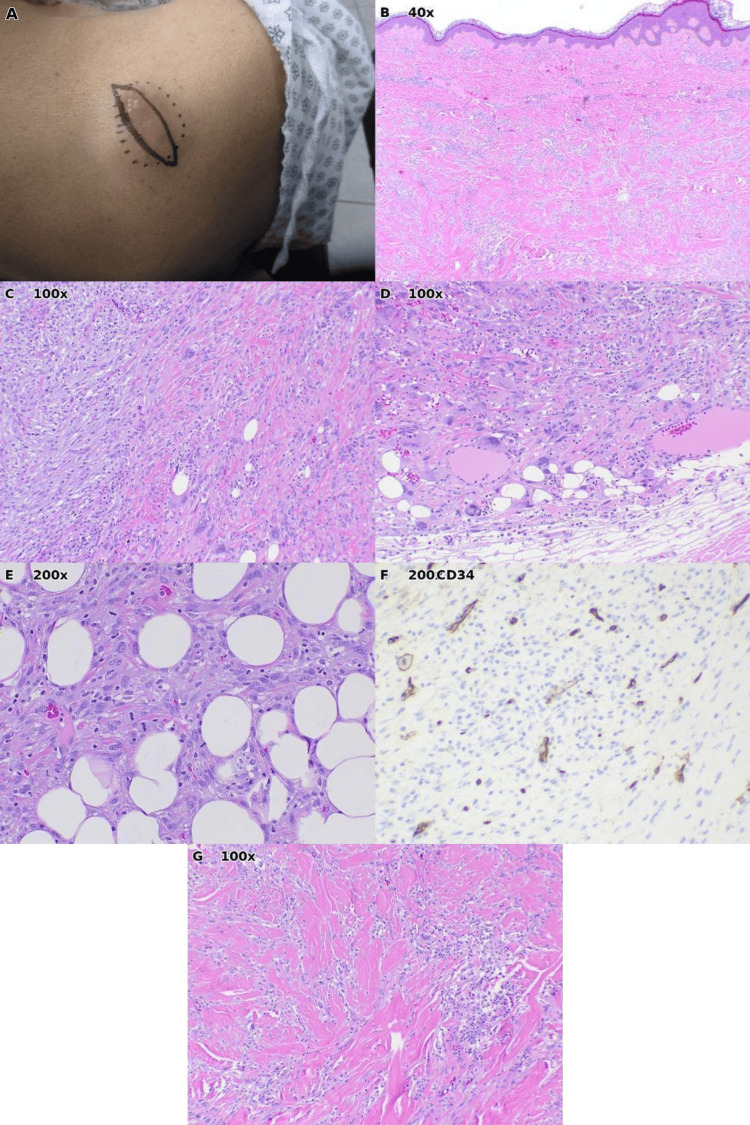
Case 4: Undifferentiated pleomorphic sarcoma of the right shoulder. (A) Clinical photograph showing a mass with elliptical surgical excision markings. (B) Low-power histopathology (H&E, ×40) demonstrating a highly cellular tumor beneath the epidermis. (C) Intermediate magnification (H&E, ×100) showing tumor cells infiltrating between adipocytes. (D) Additional view (H&E, ×100) illustrating the interface between tumor and adjacent tissue with fat entrapment. (E) High-power view (H&E, ×200) demonstrating marked cellular pleomorphism with atypical spindle and epithelioid cells. (F) Immunohistochemical stain (×200). (G) Additional view (H&E, ×100) illustrating tumor heterogeneity with dense spindle cell areas.

A 74-year-old female presented with a bump on the right shoulder that had been present for approximately two months (Figure 4A). The lesion was initially thought to represent an infectious or inflammatory process, and the patient was placed on oral doxycycline. When no improvement was noted after one month of antibiotic therapy, a surgical excision was performed to establish a definitive diagnosis.

Histopathologic examination revealed a high-grade malignancy: a pleomorphic spindle cell neoplasm characterized by marked nuclear atypia with hyperchromatic, irregularly shaped nuclei and brisk mitotic activity, including atypical mitotic figures (Figures 4B-4G). The tumor demonstrated aggressive growth with extensive infiltration into subcutaneous adipose tissue, with tumor cells surrounding and entrapping adipocytes. The pathology report indicated undifferentiated pleomorphic sarcoma (UPS), consistent with high-grade sarcoma, grade 3 of 3 according to the French Federation of Cancer Centers (FNCLCC) grading system.

The patient initially rejected the diagnosis and requested that the slides be sent to another pathologist for review. She was subsequently seen by oncology and referred to orthopedic oncology for further management, but was lost to follow-up from our institution.

## Discussion

This four-case series highlights the diagnostic pitfalls of presuming that all erythematous, inflamed nodules represent epidermal inclusion cysts. While EICs are common and benign, their clinical appearance may overlap with a spectrum of other entities, ranging from benign neoplasms such as dermatofibroma to aggressive malignancies, including amelanotic melanoma, dermatofibrosarcoma protuberans, and undifferentiated pleomorphic sarcoma.

Several clinical and dermoscopic features can aid in distinguishing EICs from their mimickers. Table 1 summarizes the classic clinical characteristics of EICs, while Table 2 outlines key distinguishing features and recommended management strategies for each entity discussed in this series.

**Table 1:** Classic features of epidermal inclusion cysts distinguish them from neoplastic mimickers. Credit: Authors.

Feature	Findings
Punctum	Visible central opening communicating with the skin surface
Mobility	Discrete, dome-shaped, freely mobile subcutaneous nodule
Contents	Keratinous, cheesy material is expressible with pressure
Histopathology	Cyst wall lined by stratified squamous epithelium with a granular layer; lumen filled with laminated keratin
Inflammation	Rupture may cause erythema and tenderness, but the cyst remains superficially mobile

**Table 2:** Clinical clues and guideline-based management for erythematous nodules mimicking epidermal inclusion cysts. Abbreviations: WLE, wide local excision; SLNB, sentinel lymph node biopsy; PDEMA, peripheral and deep en face margin assessment. Credit: Authors.

Clinical clue	Suggests	Management
Visible punctum with expressible keratin; freely mobile	Epidermal inclusion cyst	Observation or complete excision if symptomatic [1]
Dimple sign (central puckering with lateral pressure); no punctum	Dermatofibroma	Usually none; excision if symptomatic or enlarging [2]
Rapid growth; no punctum; polymorphous vessels or milky-red areas on dermoscopy	Amelanotic melanoma	Excisional biopsy with 1-3 mm margins; WLE per Breslow thickness; SLNB if ≥0.8 mm or high-risk features [5]
Slow-growing plaque evolving into nodules; reddish discoloration; induration; no punctum	Dermatofibrosarcoma protuberans	Mohs/PDEMA preferred; WLE with 2-3 cm margins if Mohs unavailable [6]
Deep, fixed mass tethered to fascia/muscle; progressive growth; no punctum	Undifferentiated pleomorphic sarcoma	MRI and image-guided core biopsy; wide en bloc excision (R0) ± radiotherapy for high-grade tumors [6]

Among these entities, the malignant mimickers pose particular diagnostic challenges. Amelanotic melanomas lack the brown-black pigmentation of conventional melanoma, making clinical recognition challenging; they represent 2-8% of all melanomas and carry a worse prognosis, partly due to delayed diagnosis [4,8]. DFSP characteristically begins as an indurated plaque that may be skin-colored, red-brown, or violaceous, slowly evolving over months to years into protuberant nodules [9,10]. This indolent growth pattern frequently leads to misdiagnosis as a cyst, keloid, or lipoma, with diagnostic delays averaging one to five years [9]. Histologically, the characteristic honeycomb infiltration of adipose tissue distinguishes DFSP from other spindle cell tumors [9,10].

Surgical management varies by entity. Dermatofibromas are benign and typically require no treatment unless symptomatic [2]. For amelanotic melanoma, the National Comprehensive Cancer Network (NCCN) guidelines recommend wide local excision with margins determined by Breslow thickness; sentinel lymph node biopsy should be considered for appropriate candidates [5]. DFSP requires margin-controlled surgery: NCCN guidelines recommend Mohs or peripheral and deep en face margin assessment (PDEMA) as preferred treatment, with lower recurrence rates than wide local excision (0-6.6% vs. 1.7-30.8%) [9]. When Mohs is unavailable, European guidelines recommend excision with 2-3 cm margins [11]. Long-term surveillance is essential, as 25% of DFSP recurrences occur beyond the typical five-year follow-up period [12]. UPS is a diagnosis of exclusion, a high-grade sarcoma lacking lineage-specific differentiation despite thorough sampling and ancillary studies. The NCCN guidelines recommend wide en bloc excision with negative margins, often combined with radiotherapy [6,13]. Table 3 summarizes histopathologic features and management strategies.

**Table 3:** Differential diagnosis for erythematous nodules mimicking epidermal inclusion cysts. Abbreviations: WLE, wide local excision; SLNB, sentinel lymph node biopsy; RT, radiotherapy; PDEMA, peripheral and deep en face margin assessment. Credit: Authors.

Entity	Clinical clues	Histopathology	Management
Epidermal inclusion cyst	Freely mobile; visible punctum; expressible keratin	Stratified squamous epithelium with granular layer; laminated keratin in lumen	Observation or complete excision if symptomatic
Dermatofibroma	Firm papule/nodule on extremities; dimple sign; no punctum; dermoscopy: central white patch, peripheral pigment network	Storiform spindle cell proliferation; epidermal hyperplasia; collagen trapping; CD34 negative or focal peripheral staining only	Usually none; excision for symptomatic or enlarging lesions
Amelanotic melanoma	Rapidly enlarging pink/red nodule; no punctum; dermoscopy: polymorphous vessels, milky-red areas	Atypical melanocytic proliferation; loss of pigment; S100 positive (97-100%); SOX10 positive	Excisional biopsy (1-3 mm margins); WLE per Breslow thickness; SLNB for tumors ≥0.8 mm
Dermatofibrosarcoma protuberans	Slow-growing plaque evolving to nodules; no punctum; reddish discoloration	Storiform spindle cells; honeycomb infiltration of subcutis; diffuse strong CD34 positivity throughout tumor	Mohs/PDEMA preferred; WLE with 2-3 cm margins if unavailable
Undifferentiated pleomorphic sarcoma	Deep fixed mass; tethered to fascia/muscle; progressive growth; no punctum	Pleomorphic spindle cells; high mitotic activity; lacks lineage-specific markers (diagnosis of exclusion)	MRI + core needle biopsy; wide en bloc excision (R0); ± adjuvant RT for high-grade

Immunohistochemistry plays a crucial role in differentiating these entities. S100 and SOX10 positivity support melanocytic lineage in amelanotic melanoma, while the distinction between peripheral or focal CD34 staining in dermatofibroma versus diffuse CD34 positivity in DFSP helps differentiate these spindle cell tumors [2,9].

This series is limited by the small number of cases and a single-institution setting. However, the diversity of pathologies encountered reinforces a key teaching point: clinicians must remain vigilant for neoplastic mimickers, particularly in settings where cysts are commonly diagnosed. When clinical features are atypical, biopsy should not be delayed.

## Conclusions

Not all inflamed nodules are cysts. Careful assessment of punctum, dimple sign, growth pattern, depth, patient medical history, and dermoscopic features enables clinicians to avoid misdiagnosis. When features are atypical, biopsy should not be delayed. Recognizing these clinical red flags and applying current guideline-based management prevents unnecessary morbidity and improves patient outcomes, particularly when aggressive malignancies masquerade as benign lesions.
